# Loss of cardiac myosin light chain kinase contributes to contractile dysfunction in right ventricular pressure overload

**DOI:** 10.14814/phy2.15238

**Published:** 2022-04-05

**Authors:** Vidhya Prasad, Nour Makkaoui, Rohan Rajan, Alisha Patel, Bipul Mainali, Pritha Bagchi, Rhea Kumar, Julia Rogers, Jake Diamond, Joshua T. Maxwell

**Affiliations:** ^1^ 12239 Division of Pediatric Cardiology Department of Pediatrics Emory University School of Medicine Atlanta Georgia USA; ^2^ Children’s Heart Research & Outcomes (HeRO) Center Children’s Healthcare of Atlanta & Emory University Atlanta Georgia USA; ^3^ Emory University College of Arts and Sciences Atlanta Georgia USA; ^4^ 12239 Department of Biochemistry Emory University School of Medicine Atlanta Georgia USA

**Keywords:** calcium, congenital heart defect, heart failure, hypertrophy, myosin light chain kinase

## Abstract

Nearly 1 in every 100 children born have a congenital heart defect. Many of these defects primarily affect the right heart causing pressure overload of the right ventricle (RV). The RV maintains function by adapting to the increased pressure; however, many of these adaptations eventually lead to RV hypertrophy and failure. In this study, we aim to identify the cellular and molecular mechanisms of these adaptions. We utilized a surgical animal model of pulmonary artery banding (PAB) in juvenile rats that has been shown to accurately recapitulate the physiology of right ventricular pressure overload in young hearts. Using this model, we examined changes in cardiac myocyte protein expression as a result of pressure overload with mass spectrometry 4 weeks post‐banding. We found pressure overload of the RV induced significant downregulation of cardiac myosin light chain kinase (cMLCK). Single myocyte calcium and contractility recordings showed impaired contraction and relaxation in PAB RV myocytes, consistent with the loss of cMLCK. In the PAB myocytes, calcium transients were of smaller amplitude and decayed at a slower rate compared to controls. We also identified miR‐200c, which has been shown to regulate cMLCK expression, as upregulated in the RV in response to pressure overload. These results indicate the loss of cMLCK is a critical maladaptation of the RV to pressure overload and represents a novel target for therapeutic approaches to treat RV hypertrophy and failure associated with congenital heart defects.

## Graphical abstract

Many congenital heart defects primarily affect the right heart causing pressure overload of the right ventricle (RV) and eventually lead to RV hypertrophy and failure. We found pressure overload of the RV induced significant downregulation of cardiac myosin light chain kinase (cMLCK), which lead to impaired contraction and relaxation in PAB RV myocytes consistent with cMLCK loss, and also found upregulation of miR‐200c, which has been shown to regulate cMLCK expression. These results indicate the loss of cMLCK is a critical maladaptation of the RV to pressure overload and represents a novel target for therapeutic approaches to treat RV hypertrophy and failure associated with congenital heart defects.

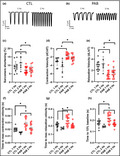

## 1 INTRODUCTION

Nearly 1% of babies born in the US will be diagnosed with a congenital heart defect (CHD). CHDs are the most common birth defect and are the number one cause of death from birth defects during the first year of life (Jenkins et al., 2007). In the pediatric population, CHDs are the leading cause of right‐ventricular (RV) hypertrophy and heart failure, especially in patients with defects like pulmonary stenosis, hypoplastic left heart syndrome and tetralogy of Fallot (Guihaire et al., 2012). In these complex CHDs, the RV is subject to pressure overload leading to hypertrophy and, when the heart is under sustained stress, the hypertrophic response can evolve into decompensated heart failure. Considerable work has been done in the pressure‐overloaded left ventricle uncovering many of the maladaptation in protein expression, post‐translational modifications, calcium (Ca^2+^) handling, and contractility of left ventricle cardiac myocytes. However, much of this basic and clinical research has focused on the LV, and our understanding of the adaptions of the RV to pressure overload and the cellular and molecular mechanisms of these adaptions are critically lacking.

Therapeutic options for RV failure are limited and much of the basic and clinical research has historically focused on the LV. There has been considerable data emerge on the differences between the right and left ventricle in development, structure, function, and pathologies (Strniskova et al., 2006; Wang et al., 2006), which have consequences for the evaluation and treatment strategy for patients (Winter et al., 2009). Therefore, generalizing heart failure treatments and attempting to extrapolate data and experience from left‐ventricular (LV) failure to RV failure is futile. Furthermore, pediatric myocardium responds to pharmacological treatments differently than in adults, and therapies effective in LV failure have been proven ineffective or even harmful in RV failure (Schranz & Voelkel, 2016). For example, standard left ventricular heart failure therapies such as β‐blockers, ACE‐inhibitors, AngII receptor blockers have little to no effect on RV failure in the setting of CHDs (Hsu et al., 2010; Shaddy et al., 2007; Szymanski et al., 2009; Winter et al., 2009). Current therapeutics for RV failure including pharmacological agents, further surgical or mechanical interventions, and eventually heart transplantation at the end‐stage of HF make the clinical management of patients with RV failure both costly and challenging (Winter et al., 2009). While basic research has provided initial insights into the disease mechanisms of RV pressure overload (Assad & Hemnes, 2015; Gomez‐Arroyo et al., 2013; Piao et al., 2010; Reddy & Bernstein, 2015; Urashima et al., 2008), more research is needed to explore the molecular mechanisms of pressure overload‐induced RV dysfunction and to develop new therapeutic strategies to treat or reverse RV failure (Voelkel et al., 2006).

Using a rat model of RV pressure overload in which the pulmonary artery is surgically banded (PAB), we examined changes in cardiac myocyte protein expression as a result of pressure overload with mass spectrometry 4 weeks post‐banding. We found pressure overload of the RV induced significant downregulation of cardiac myosin light chain kinase (cMLCK) in the PAB myocytes through RV proteomic analysis. cMLCK is an abundantly expressed protein that is critical for normal contractile function of cardiac myocytes and loss of cMLCK has been shown to play a role in the development of left ventricular hypertrophy (Chan et al., 2008; Gu et al., 2010; Hu et al., 2019; Massengill et al., 2016; Warren et al., 2012). We measured isolated cardiac myocyte calcium handing and contractility in normal and PAB RV myocytes and found impaired contraction and relaxation in PAB RV myocytes, along with dysfunctional diastolic calcium handling. We also identified miR‐200c as upregulated in the RV in response to pressure overload and may control expression of cMLCK. These results indicate the loss of cMLCK is a critical maladaptation of the RV to pressure overload and represents a novel target for therapeutic approaches to treat RV hypertrophy and failure associated with congenital heart defects.

## 2 METHODS

### 2.1 Solutions and chemicals

All chemicals and reagents were purchased from Sigma‐Aldrich unless otherwise stated. Fluo‐4/AM Ca^2+^ indicator dye was purchased from Molecular Probes/Invitrogen. Tyrode's solution contained (in mM) 138 NaCl, 4 KCl, 2 CaCl_2_, 1 MgCl_2_, 10 glucose, and 10 HEPES; pH 7.4 with NaOH. A cMLCK inhibitor, ML‐7 (Tocris), was used at a concentration of 10 µM in normal Tyrode's solution.

### 2.2 Rat pulmonary artery banding model

All animal experiments were performed with the approval of the Institutional Animal Care and Use Committee of Emory University and conform to the guidelines from the NIH Guide for the Care and Use of Laboratory Animals. Male pediatric (3–4 weeks old) Sprague‐Dawley rats (~120–150 g) were obtained from Envigo (RRID:RGD_737903). All rats exhibited normal RV function on echocardiography at the time of pulmonary artery banding (PAB) surgery (4–5 weeks old). Rats were anesthetized with 2% isoflurane until no response from toe pinch reflex, USP (Piramal Healthcare), and a limited left thoracotomy was performed to expose the pulmonary artery (PA). The PA was dissected from the aorta and partially ligated over an 18‐gauge angiocatheter. The sizer was then promptly removed to allow for antegrade flow through the banded area, and thoracotomy performed was closed under positive pressure ventilation to evacuate pleural air (*n* = 18). Sham operated control (CTL) animals underwent the same procedure without banding the PA (*n* = 16). Transthoracic echocardiography was performed prior to surgery and at 4 weeks post‐banding using a Vevo 2100 digital high‐frequency ultrasound system (FujiFilm Visualsonics) equipped with a probe (MS250) suited for rat imaging. Tricuspid annular plane systolic excursion (TAPSE) was measured in the apical four‐chamber view in M‐mode. RV end diastolic diameter (RVEDD) was measured from the interventricular septum to the RV free wall in the apical four‐chamber view in B‐mode. RV wall thickness during diastole (RVWT;d) was measured in the two‐dimensional short‐axis view in M‐mode. As previously shown, this surgery produces severe right ventricular dysfunction within two weeks post‐banding (Maxwell et al., 2019; Trac et al., 2018). Right ventricular dysfunction was confirmed as at least a 40% reduction in TAPSE. TAPSE, RVEDD, and RVWT;d values for all individual animals used in this study are shown in Figure S1. Hearts were extracted for analysis at 4 weeks post‐banding for the PAB group or 4 weeks post‐sham surgery for the CTL group.

### 2.3 Myocyte isolation

Single right ventricular myocytes were isolated from CTL and PAB rats. Rats were anesthetized with ketamine (0.1 mg/g) and xylazine (0.01 mg/g) until loss of toe pinch reflex, and hearts were excised and mounted on a Langendorff apparatus. Hearts were retrogradely perfused with nominally Ca^2+^‐free Tyrode's solution for 5 min followed by Minimal Essential Medium Eagle (MEM) solution containing 20 µM Ca^2+^ and 22.5 µg/ml Liberase Blendzyme TH (Roche Applied Science) for 25–45 min at 37°C. The right ventricular free wall was removed from the heart, minced, filtered and washed in MEM solution containing 50 µM Ca^2+^ and 10 mg/ml bovine serum albumin. Isolated cells were kept in MEM solution with 50 µM Ca^2+^ at room temperature (22–24°C) until indicator dye loading and subsequent experimentation. For some experiments, the cMLCK inhibitor ML‐7 (Tocris) or the PKA inhibitor Rp‐cAMPS (Santa Cruz) was used. Myocytes were treated for 5 min with 10 µM ML‐7 or 100 µM Rp‐cAMPS in normal Tyrode's solution before data acquisition.

### 2.4 Mass spectrometry

300 µL of urea lysis buffer (8 M urea, 10 mM Tris, 100 mM NaH_2_PO_4_, pH 8.5), including 3 µL (100x stock) HALT(‐EDTA) protease and phosphatase inhibitor cocktail (Pierce) was added to the cell pellets. Samples were sonicated (Sonic Dismembrator, Fisher Scientific) three times for 5 sec each with 5 sec intervals of rest at 30% amplitude to disrupt nucleic acids and were subsequently centrifuged at 4°C. Protein concentration was determined by the bicinchoninic acid (BCA) method, and samples were frozen in aliquots at −80°C. Protein homogenates (100 µg) were treated with 1 mM dithiothreitol (DTT) at room temperature for 30 min, followed by 5 mM iodoacetimide at room temperature for 30 min in the dark. Protein samples were digested with 1:100 (w/w) lysyl endopeptidase (Wako) at room temperature for overnight. Next day, samples were diluted with 50 mM NH_4_HCO_3_ to a final concentration of less than 2 M urea and were further digested overnight with 1:50 (w/w) trypsin (Promega) at room temperature. Resulting peptides were desalted with HLB column (Waters) and were dried under vacuum.

The data acquisition by LC‐MS/MS protocol was adapted from a published procedure (Seyfried et al., 2017) and was performed by the Integrated Proteomics Core Facility at Emory University. Derived peptides were resuspended in 100 µL loading buffer (0.1% trifluoroacetic acid). Peptide mixtures (2 µL) were separated on a self‐packed C18 (1.9 µm, Dr. Maisch) fused silica column (15 cm × 100 µm internal diameter (ID); New Objective) attached to an EASY‐nLC™ 1200 system and were monitored on a Q‐Exactive Plus Mass Spectrometer (ThermoFisher Scientific). Elution was performed over a 56 min gradient at a rate of 700 nL/min (buffer A: 0.1% formic acid in water, buffer B: 0.1% formic acid in acetonitrile): The gradient started with 1% buffer B and went to 40% in 56 min, then increased from 40% to 99% within 1 min and finally staying at 99% for 3 min. The mass spectrometer cycle was programmed to collect one full MS scan followed by 20 data dependent MS/MS scans. The MS scans (400–1600 *m*/*z* range, 1 × 10^6^ AGC target, 100 ms maximum ion time) were collected at a resolution of 70,000 at *m*/*z* 200 in profile mode. The HCD MS/MS spectra (2 *m*/*z* isolation width, 28% collision energy, 1 × 10^5^ AGC target, 50 ms maximum ion time) were acquired at a resolution of 17,500 at *m*/*z* 200. Dynamic exclusion was set to exclude previously sequenced precursor ions for 20 s within a 10 ppm window. Precursor ions with +1, and +7, or higher charge states were excluded from sequencing.

Label‐free quantification analysis was adapted from a published procedure (Seyfried et al., 2017). Spectra were searched using the search engine Andromeda, integrated into MaxQuant, against rat Uniprot/Swiss‐Prot database (8097 target sequences). Methionine oxidation (+15.9949 Da), asparagine and glutamine deamidation (+0.9840 Da) and protein N‐terminal acetylation (+42.0106 Da) were variable modifications (up to five allowed per peptide); cysteine was assigned as fixed carbamidomethyl modification (+57.0215 Da). Only fully tryptic peptides with up to two miscleavages were considered in the database search. A precursor mass tolerance of ±20 ppm was applied before mass accuracy calibration and ±4.5 ppm after internal MaxQuant calibration. Other search settings included a maximum peptide mass of 6,000 Da, a minimum peptide length of six residues and 0.05‐Da tolerance for high resolution MS/MS scans. The FDR for peptide spectral matches, proteins and site decoy fraction was set to 1%. Quantification settings were as follows: match full MS1 peaks between runs; use a 0.7‐min retention time match window after an alignment function was found with a 20‐min retention time search space. The LFQ algorithm in MaxQuant was used for protein quantitation. The quantitation method considered only razor and unique peptides for protein level quantitation. Data was prepared for presentation using Perseus software. The mass spectrometry proteomics data have been deposited to the ProteomeXchange Consortium via the PRIDE partner repository with the dataset identifier PXD027427.

### 2.5 Western blot

Isolated rat right ventricular myocytes were lysed in lysis buffer (Mammalian Cell Lysis Kit, Sigma Aldrich). The protein concentration of the lysate was determined with the BCA Protein Assay (Thermo Fisher Scientific). Approximately 30 μg of lysate was mixed with 4× sample buffer, boiled for 5 min, and resolved on 7.5% Tris‐Glycine stain‐free SDS‐PAGE (Bio‐Rad). Stain‐free gels were activated with UV light for 5 min after SDS‐PAGE to allow for visualization of total protein and eventual signal normalization. After transfer to nitrocellulose membranes, the membranes were incubated with primary antibody followed by incubation with HRP‐conjugated secondary antibodies. Primary antibodies used were anti‐cardiac myosin light chain kinase (Invitrogen, PA5‐100908, 1:1000, RRID:AB_2850401), anti‐myosin light chain 2v (Invitrogen, 10906‐1‐AP, 1:1000, RRID:AB_2147453), and anti‐phospho‐myosin light chain 2v (Ser15) (Invitrogen, PA5‐104265, 1:1000, RRID:AB_2816014). The secondary antibody used was goat‐anti‐rabbit HRP‐conjugated (Bio‐Rad, #170‐6515, 1:1000, RRID:AB_11125142). Visualization was accomplished using ECL reagents (Bio‐Rad) and Bio‐Rad ChemiDoc XRS+. Stain‐free gel technology provided total protein for each lane and allowed for normalization of the antibody signal to the total protein loaded for each sample.

### 2.6 Quantitative real‐time PCR

Total RNA was extracted from myocyte cell homogenates using TRIzol reagent (Invitrogen) per manufacturer's protocol, followed by DNase I treatment and first‐strand cDNA synthesis with M‐MLV reverse transcriptase (Invitrogen) primed by random hexamers and oligo(dT)18. Real‐time PCR was then performed on the StepOne Plus System (Applied Biosystems) based on SYBR Green fluorescence detection of PCR products. The following primer sequences were used (rat species sequences):

cMLCK: forward 5′‐CCAACGTGTCCCTAATCTACAA‐3’ and reverse 5’‐CCAAGCTCCTGACTCCATAAA‐3′

GAPDH: forward 5′‐ACTCCCATTCTTCCACCTTTG‐3’ and reverse 5’‐CCCTGTTGCTGTAGCCATATT‐3′

rno‐miR‐200c‐3p Qiagen Catalog #YP00205505 and U6 snRNA Qiagen Catalog #YP00203907.

### 2.7 Sarcomere shortening measurements

Cell shortening was measured with an IonOptix Myocyte Calcium Photometry and Contractility System connected to an Olympus IX81 microscope. Sarcomere shortening was measured by Fourier analysis of the myocyte image in real time and analyzed with IonWizard software.

### 2.8 Intracellular Ca^2+^ measurements

Confocal microscopy (Fluoview 1000, Olympus Corporation) was used to image Ca^2+^ sparks, with excitation at 488 nm and emission collected at >500 nm. Cardiac myocytes were loaded with 20 µm fluo‐4/AM for 20 min at room temperature, followed by a 20 min wash in 0 Ca^2+^ Tyrode's at room temperature. Ca^2+^ spark measurements were acquired from intact myocytes perfused with Tyrode's solution during rest after 1 Hz stimulation in line scan mode at 2 ms/line with a pixel size of 0.155 µm. All fluorescent signals were background subtracted. Changes in [Ca^2+^]_i_ are expressed as ΔF/F0, where ΔF is the change in fluorescence (measured fluorescence [F] – F0) and F0 is resting baseline fluo‐4 fluorescence. Calcium release synchrony analysis was performed as previously described (Heinzel et al., 2002, 2008). Briefly, repetitive line scans were stacked yielding line scan images that were segmented according to the onset of the whole‐line averaged Ca^2+^ transients, and 6–10 sequential beats were averaged. The resulting time‐averaged line scan image of a Ca^2+^ transient therefore allowed identification of cell regions with consistently early or delayed Ca^2+^ release upon depolarization, independent of potential beat‐to‐beat variability in Ca^2+^ release. The onset of the whole‐line averaged Ca^2+^ transient was set as t0. Intensity fluorescence values (F) along the scan line were normalized to the fluorescence intensity at diastole (F0; last 50 ms of diastole prior to t0), and each line of the time‐averaged line scan image was then divided by the averaged F0 line. F50 was defined as the half‐maximum of the normalized overall peak Ca^2+^‐dependent fluorescence. Sections of the line where F did not reach F50 within 200 ms were excluded from analysis. For all Ca^2+^ imaging and contractility experiments, cells were placed on laminin‐coated coverslips. Action potentials and global Ca^2+^ transients were elicited by electrical field stimulation using a pair of platinum electrodes. Experiments were conducted at room temperature (22–24°C). Sparks were detected and analyzed by Sparkmaster (Picht et al., 2007).

### 2.9 Membrane staining

Sarcolemma and t‐tubular membranes were visualized with the membrane‐bound fluorescent probe di‐8‐ANEPPS (Molecular Probes/Life Technologies) by two‐dimensional confocal microscopy. Cells were loaded for 15 min with di‐8‐ANEPPS (5 μm) in Tyrode solution and the indicator was excited at 488 nm, and emission was measured at >600 nm. Transverse tubule analysis was performed using the ImageJ/Fiji plugin TTorg (Pasqualin et al., 2015).

### 2.10 Ca^2+^ measurement presentation

Confocal line scan data and fluorescence traces are presented as individual observations representative of multiple recordings or as the average of multiple recordings. Fluorescence traces were background subtracted and plotted as *F*/*F*_0_, where *F*_0_ is the basal fluorescence in resting cells at the beginning of a recording or diastolic fluorescence in stimulated cells. Ca^2+^ signal amplitudes are expressed as ∆*F*/*F*_0_, where ∆*F* = *F* − *F*_0_.

### 2.11 Statistical analysis

Data are presented as mean ± SD unless otherwise noted in the legend. Statistical analysis was performed using unpaired *t*‐test (GraphPad Prism, v9.2.0) as noted. Differences were considered statistically significant at *p* < 0.05.

## 3 RESULTS

**FIGURE 1:**
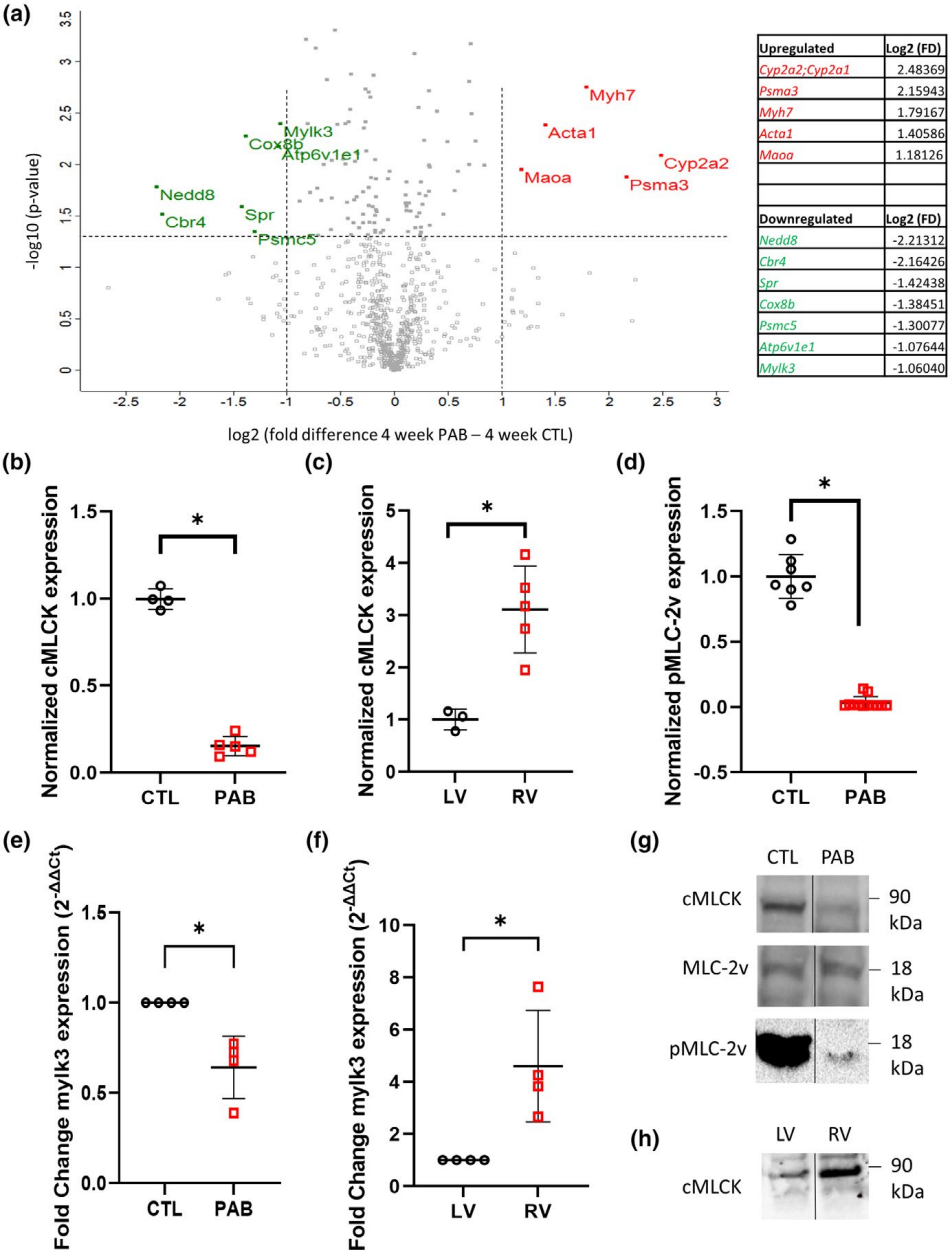
cMLCK is downregulated in RV myocytes from PAB animals. (a) Volcano plot of proteins identified and quantified by mass spectrometry in the RV myocytes from PAB versus CTL animals (positive log2(fold change) indicates higher expression in PAB myocytes). Each point represents the difference in expression (*x*‐axis) between the groups and the associated significance of this change (*y*‐axis) by independent unpaired samples *t*‐tests. Proteins that are significantly (±1.3‐fold, *p* < 0.05) upregulated (red) or downregulated (green) are identified with gene labels (*n* = 4 hearts each group). Inset table shows the significantly upregulated (red) and downregulated (green) protein and the log2 (fold difference) values for each. (b) Protein expression graphs of quantified Western blot data for cMLCK in CTL RV versus PAB RV (*p* < 0.001, unpaired *t*‐test, *n* = 4 CTL hearts, 5 PAB hearts) and in (c) CTL LV versus CTL RV (*p* < 0.001, unpaired *t*‐test, *n* = 3 LV, 5 RV), and for (d) pMLC‐2v in CTL versus PAB RV (*p* < 0.001, unpaired *t*‐test, *n* = 7 CTL, 9 PAB). (e) Real time RT‐PCR mRNA expression data for mylk3 gene from CTL RV versus PAB RV (*p* = 0.006, unpaired *t* test, *n* = 4 CTL, 4 PAB) and (f) CTL LV versus CTL RV (*p* = 0.02, unpaired *t*‐test, *n* = 4 CTL, 4 PAB). (g, h) Representative Western blots for cMLCK, MLC‐2v, and pMLC‐2v. Gel lanes were arranged from original blot for clarity (*p < 0.05, unpaired *t*‐test). All N’s represent individual hearts or heart tissues from individual animals

Even after successful repair of many congenital heart defects, right ventricle (RV) pressure overload remains in some patients and eventually impairs RV function and influences long‐term mortality and morbidity. Although compensated hypertrophy develops initially, ultimately RV failure will occur. The mechanisms underlying the progression from compensated to decompensated RV hypertrophy have not been well defined. To recapitulate the human disease, we utilized a juvenile animal model of right ventricular pressure overload created by partial pulmonary artery banding in juvenile rats. We first sought to identify the changes in right ventricular cardiac myocyte protein expression as a result of pressure overload on the juvenile right ventricle. To enrich for myocyte‐specific proteins, cardiac myocytes were isolated and purified from sham operated control (CTL) and pulmonary artery banded (PAB) right ventricles and subjected to mass spectrometry analysis 4 weeks post‐surgery. Tandem MS/MS analysis revealed several key changes in protein expression between CTL and PAB RV cardiac myocytes (Figure 1a). Upregulation of markers of cardiac hypertrophy including *Myh7* and *Acta1* were observed in the RV myocytes of PAB animals, validating our surgical model (Köhler et al., 2013; Lowes et al., 1997). Interestingly, cardiac myosin light chain kinase (cMLCK) was significantly downregulated in RV cardiac myocytes from PAB animals compared to CTL. This finding was verified with Western blot (Figure 1b), and RT‐PCR was used to show the mRNA (*mylk3*) was also downregulated in PAB animals compared to CTL (Figure 1e). Previous studies have shown higher expression of cMLCK in the RV compared to the left ventricle (Warren et al., 2012). In agreement with this, we also found significantly higher levels of cMLCK protein (Figure 1c) and *mylk3* mRNA (Figure 1f) in the RV compared to the left ventricle in our CTL animals. We also observed decreased myosin light chain (MLC‐2v) phosphorylation at Ser15 in PAB RV myocytes compared to CTL (Figure 1d), consistent with a loss of cMLCK. Representative Western blots for the above data are shown in Figure 1g,h.

**FIGURE 2:**
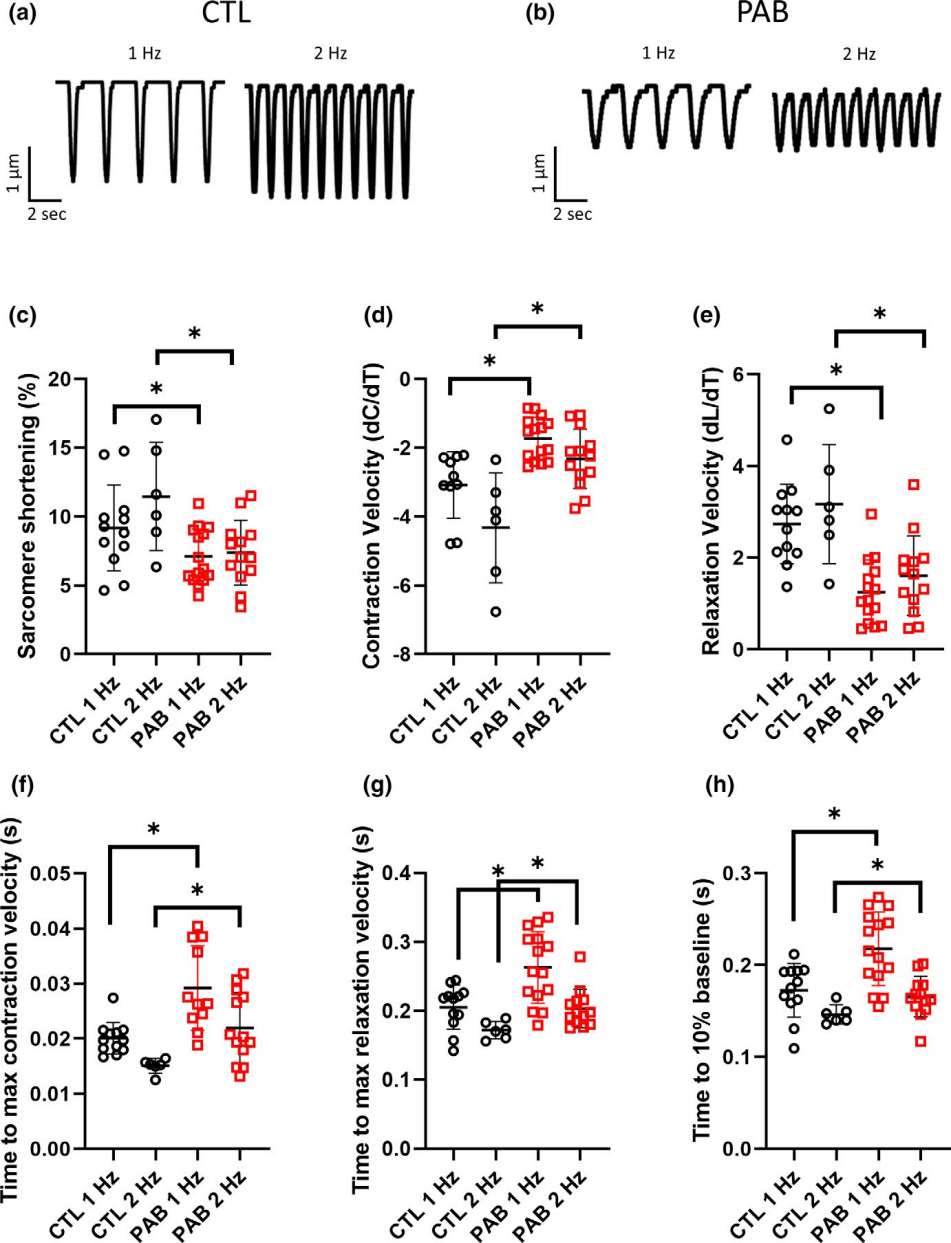
Single RV myocytes from PAB rats show impaired contraction and relaxation. Representative sarcomere shortening traces from (a) CTL and (b) PAB RV myocytes at 1 and 2 Hz stimulation frequencies. Summary graphs of sarcomere shortening parameters including (c) sarcomere shortening % (1 Hz: *p* = 0.047, 2 Hz: *p* = 0.01, unpaired *t*‐test), (d) contraction velocity (1 Hz: *p* < 0.001, 2 Hz: *p* = 0.002, unpaired *t*‐test), (e) relaxation velocity (1 Hz: *p* < 0.001, 2 Hz: *p* = 0.006, unpaired *t*‐test), (f) time to max contraction (1 Hz: *p* < 0.001, 2 Hz: *p* = 0.02, unpaired *t*‐test), (g) time to max relaxation (1 Hz: *p* = 0.003, 2 Hz: *p* = 0.02, unpaired *t*‐test), and (h) time to 10% baseline (1 Hz: *p* = 0.003, 2 Hz: *p* = 0.048, unpaired *t*‐test). (*n* = 13, CTL 1 Hz; *n* = 6, CTL 2 Hz; *n* = 15, PAB 1 Hz; *n* = 13 PAB 2 Hz) (*p < 0.05, unpaired *t* test)

**FIGURE 3:**
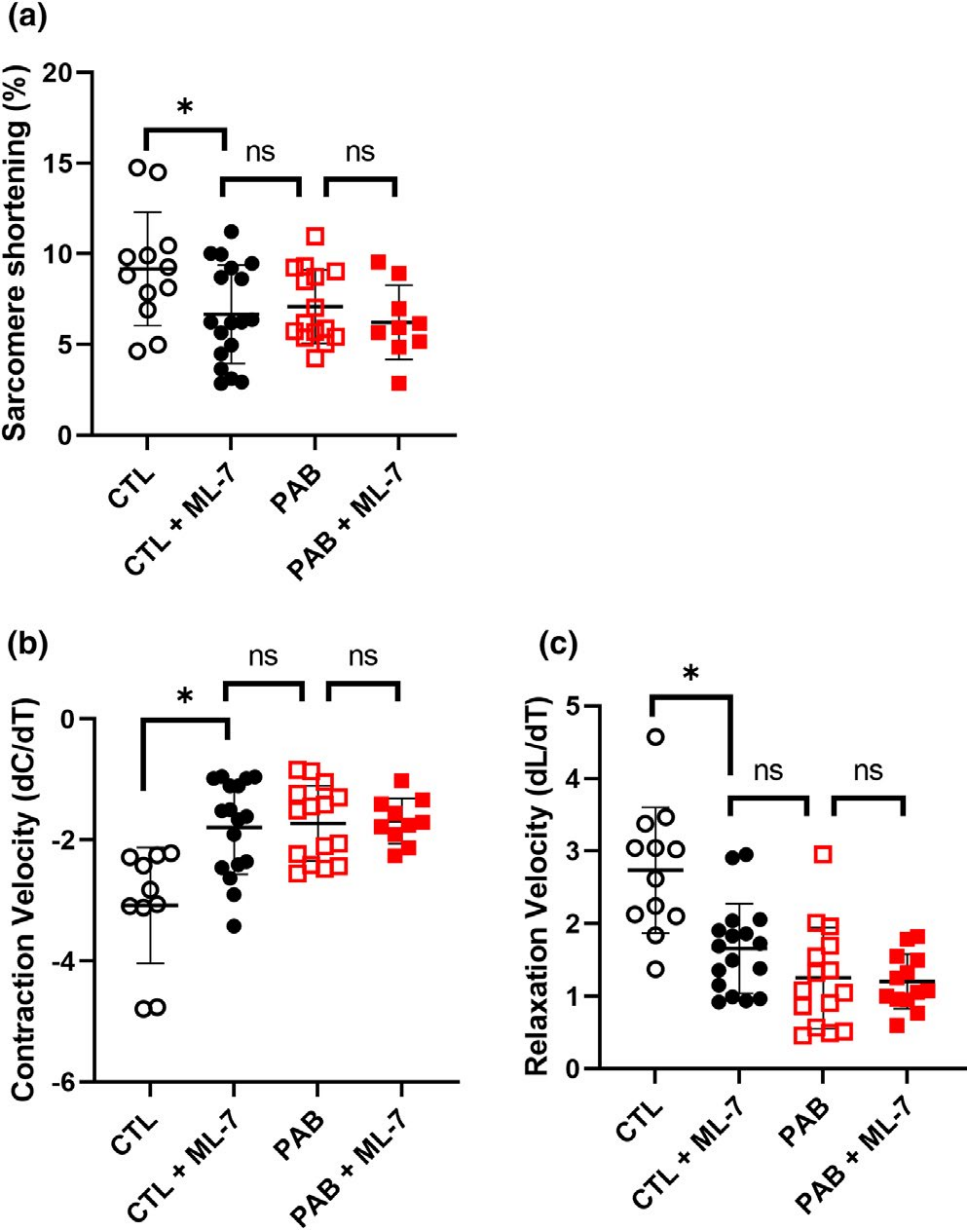
cMLCK inhibitor significantly affects sarcomere contraction and relaxation in CTL but not PAB RV myocytes. CTL and PAB RV myocytes were acutely treated with a cMLCK inhibitor, ML‐7 (5 min, 10 µM) and sarcomere shortening recordings were performed with 1 Hz stimulation frequency. Summary graphs of sarcomere shortening parameters including (a) sarcomere shortening % (CTL vs. CTL + ML‐7, *p* = 0.027; CTL + ML‐7 vs. PAB, *p* = 0.62; PAB vs PAB + ML‐7, *p* = 0.32; unpaired *t*‐test), (b) contraction velocity (CTL vs. CTL + ML‐7, *p* < 0.001; CTL + ML‐7 vs. PAB, *p* = 0.80; PAB vs. PAB + ML‐7, *p* = 0.86; unpaired *t*‐test), and (c) relaxation velocity (CTL vs. CTL + ML‐7, *p* < 0.001; CTL + ML‐7 vs. PAB, *p* = 0.09; PAB vs. PAB + ML‐7, *p* = 0.82; unpaired *t*‐test), (*n* = 12, CTL; *n* = 17, CTL + ML‐7; *n* = 16, PAB; *n* = 10 PAB + ML‐7) (*p < 0.05, unpaired *t* test)

Loss of cMLCK expression has been reported in left ventricular hypertrophy and heart failure as a result of pressure overload (Chan et al., 2008; Gu et al., 2010; Sweeney & Stull, 1986; Warren et al., 2012). Functionally, this resulted in decreased fractional shortening and rates of contraction and relaxation in isolated cardiac myocytes due to decreased myosin light chain (MLC) phosphorylation (Massengill et al., 2016). However, the functional effects of decreased cMLCK expression in the RV have yet to be explored. Utilizing our PAB animal model, we first examined the effect of pressure‐overload on the contractility parameters of primary right ventricular myocytes. Single cell contractility measurements were performed using CTL and PAB myocytes isolated from hearts 4 weeks post‐surgery (sham or PAB). Cells were paced at frequencies of 1 and 2 Hz. Representative traces of sarcomere length measurements are shown in Figure 2a,b. RV myocytes from PAB hearts showed significantly decreased contractility (Figure 2c), contraction velocity (Figure 2d), relaxation velocity (Figure 2e), at both 1 and 2 Hz stimulation frequencies. Additionally, the time to max contraction (Figure 2f) and max relaxation (Figure 2g) velocities were also significantly longer in PAB RV myocytes compared to control, along with the time to 10% baseline (Figure 2h), at both 1 and 2 Hz stimulation frequencies. Consistent with the findings in LV myocytes, pressure overload resulted in severe defects in both contraction and relaxation of RV myocytes. To examine the role of cMLCK in regulating these changes, we utilized a known inhibitor of MLCK, ML‐7 (Gu et al., 2010). Treatment of CTL myocytes with 10 µM ML‐7 resulted in significantly decreased contractility (Figure 3a), contraction velocity (Figure 3b) and relaxation velocity (Figure 3c), to levels similar to as seen in PAB myocytes. PAB RV myocytes treated with ML‐7 showed no further decreases in contractility, contraction velocity, or relaxation velocity (Figure 3a–c). CTL and PAB myocytes were also treated with a PKA inhibitor, Rp‐cAMPS. Inhibition of PKA with Rp‐cAMPS had no significant effect on the contractility, contraction velocity, or the relaxation velocity in either the CTL or PAB RV myocytes (Figure S2).

**FIGURE 4:**
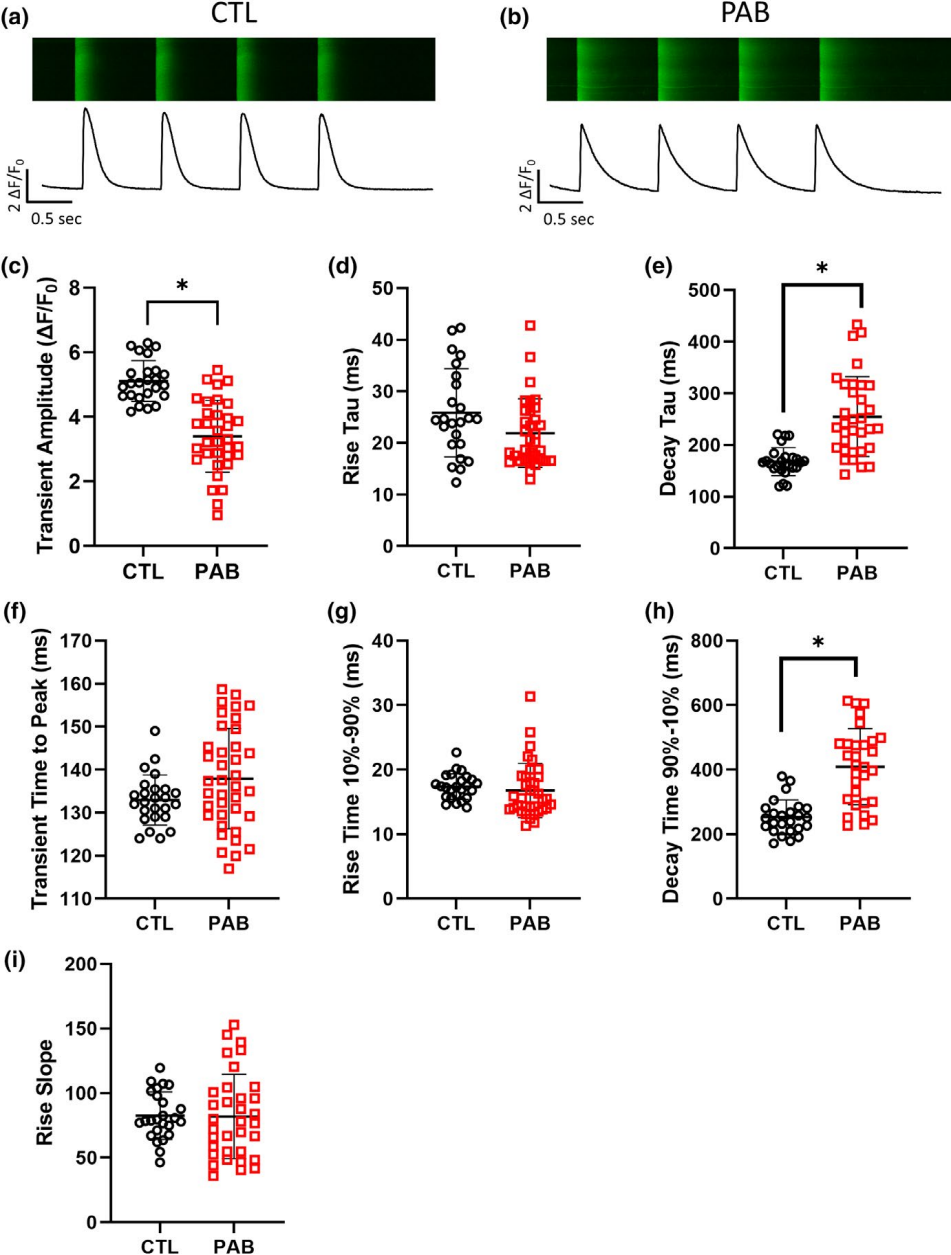
PAB RV myocytes show impaired diastolic Ca^2+^ handling compared to CTL. Representative traces of cytosolic fluo‐4 fluorescence in (a) CTL and (b) PAB RV myocytes during 1 Hz stimulation. Summary graphs of Ca^2+^ transient parameters including (c) transient amplitude (*p* < 0.001, unpaired *t*‐test), (d) rise tau (*p* = 0.06, unpaired *t*‐test), (e) decay tau (*p* < 0.001, unpaired *t*‐test), (f) transient time to peak (*p* = 0.053, unpaired *t*‐test), (g) rise time 10%–90% (*p* = 0.50, unpaired *t*‐test), (h) decay time 90%–10% (*p* < 0.001, unpaired *t*‐test), and (i) rise slope (*p* = 0.92, unpaired *t*‐test). (*n* = 25 CTL, *n* = 34 PAB) (*p < 0.05, unpaired *t* test)

**FIGURE 5:**
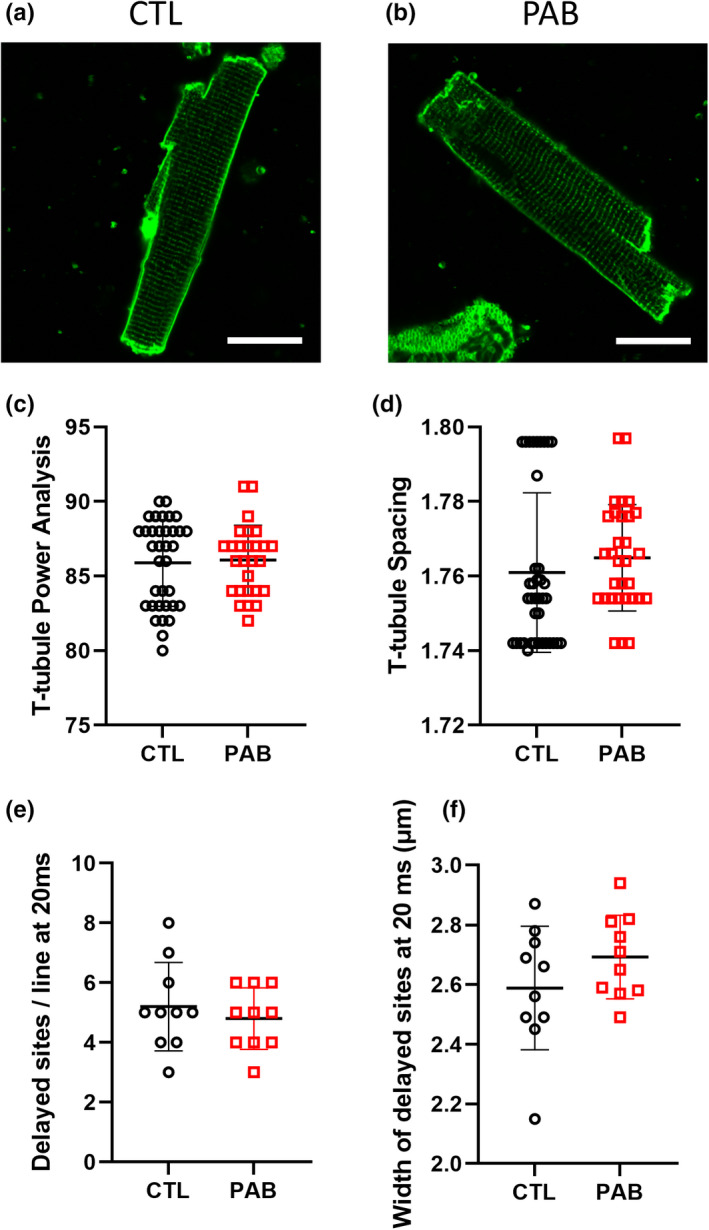
CTL and PAB RV myocytes show similar transverse tubule structure. Representative image of (a) CTL and (b) PAB myocyte stained with di‐8‐ANEPPS (Scale bar = 25 µm). Summary graphs of the quantification of *t*‐tubule structure including (c) *t*‐tubule power analysis (*p* = 0.78, unpaired *t*‐test) and (d) *t*‐tubule spacing (*p* = 0.38, unpaired *t*‐test) in CTL and PAB RV myocytes (*n* = 35 CTL, *n* = 27 PAB). Summary graphs of calcium release synchrony analysis showing both the (e) number and (f) size of regions with delayed Ca^2+^ release (F < F50) quantified 20 ms after the onset of the overall Ca^2+^ transient (*n* = 10 CTL, *n* = 10 PAB)

**FIGURE 6:**
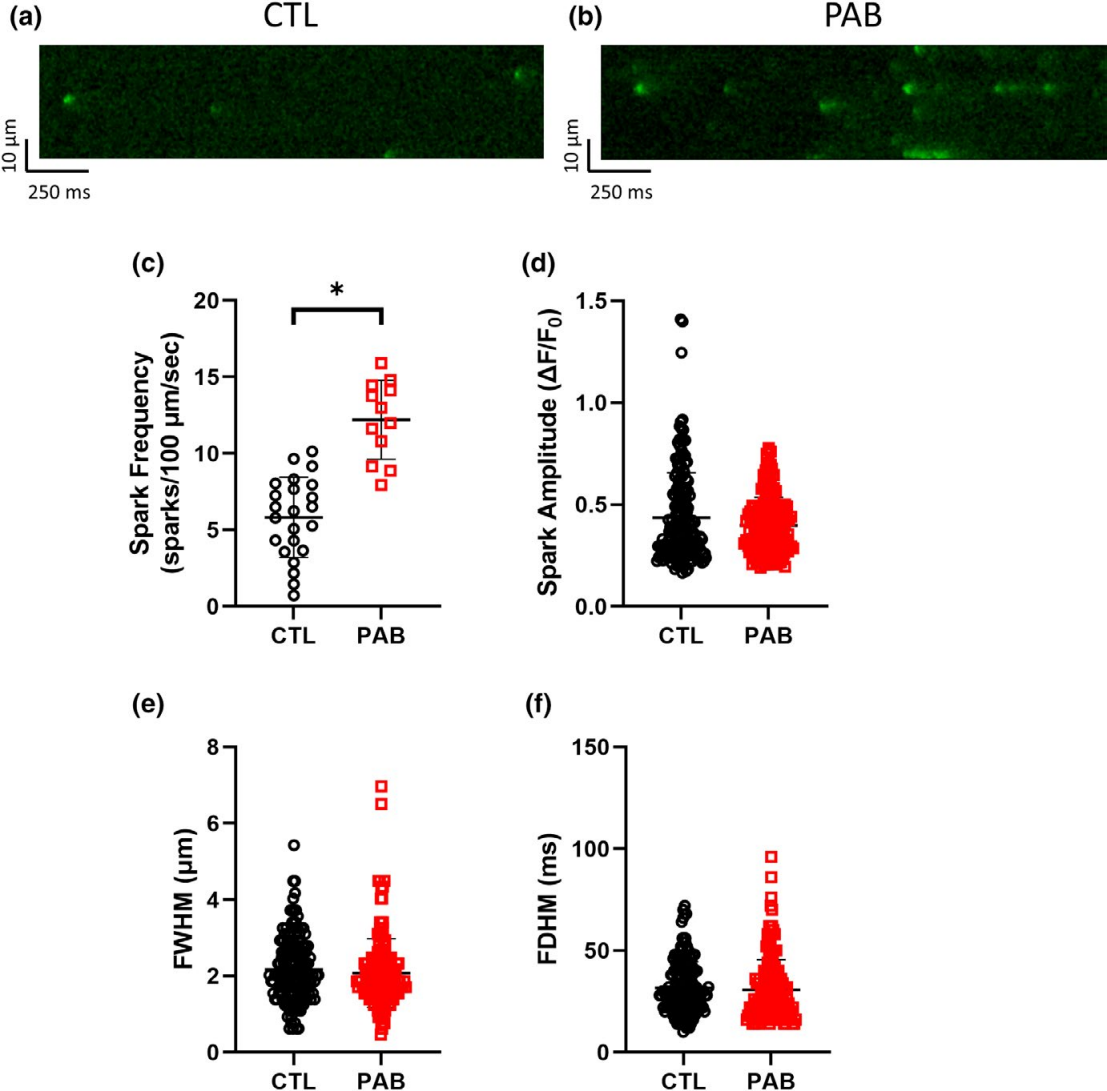
PAB RV myocytes show increased Ca^2+^ spark frequency compared to CTL. Representative traces of cytosolic fluo‐4 fluorescence in (a) CTL and (b) PAB RV myocytes after 1 Hz stimulation. Summary graphs of Ca^2+^ spark parameters including (c) spark frequency (*p* < 0.001, unpaired *t*‐test), (d) spark amplitude (*p* = 0.066, unpaired *t*‐test), (e) full width at half max (FWHM) (*p* = 0.32, unpaired *t*‐test), and (f) full duration at half max (FDHM) (*p* = 0.45, unpaired *t*‐test) (*n*_cells_ = 23 CTL, *n*_cells_ = 12 PAB; *n*_sparks_ = 180 CTL, *n*_sparks_ = 171 PAB) (*p < 0.05, unpaired *t* test)

While the loss of cMLCK expression may explain PAB myocyte contractility impairment, alterations in calcium (Ca^2+^) handling could also be playing a role. To test this, we next characterized cardiac myocyte Ca^2+^ handling in CTL and PAB RV myocytes using confocal microscopy and intracellular Ca^2+^ recordings. Representative line scans of fluo‐4/AM fluorescence are shown in Figure 4a,b from CTL and PAB animals, respectively. PAB myocytes exhibited significantly decreased Ca^2+^ transient amplitude (Figure 4c), Ca^2+^ transient decay tau (Figure 4e), and Ca^2+^ transient decay time (Figure 4h) when compared to control cells. Interestingly, we did not observe any differences in calcium release kinetics between CTL and PAB RV myocytes including rise tau (Figure 4d), time to peak (Figure 4f), rise time 10%–90% (Figure 4g) and rise slope (Figure 4i). These data indicate that Ca^2+^ release kinetics during systole are not significantly affected by pressure overload but the Ca^2+^ re‐uptake kinetics during diastole are dysregulated in PAB myocytes. Additionally, cardiac myocyte sarcolemmal membranes were with stained with Di‐8‐ANEPPS to visualize and quantify transverse tubule (t‐tubule) structures in CTL (Figure 5a) and PAB (Figure 5b) RV myocytes. T‐tubule analysis using TTorg revealed power (Figure 5c) and spacing data (Figure 5d) were not significantly different between CTL and PAB RV myocytes. Calcium release synchrony analysis was performed and showed no significant difference in the number (Figure 5e) or the size (Figure 5f) of delayed sites of calcium release between CTL and PAB RV myocytes. These data indicate the t‐tubules and the overall structure of the Ca^2+^ release mechanism was still intact 4 weeks after pressure overload. These data also are in correlation with the Ca^2+^ handling data showing no significant differences in Ca^2+^ release kinetics between CTL and PAB RV myocytes. Alternatively, the dysregulated Ca^2+^ re‐uptake kinetics in PAB myocytes observed in Figure 4 above could be affected by diastolic Ca^2+^ release events. Therefore, we measured Ca^2+^ spark parameters in CTL and PAB RV myocytes to further investigate the effect of pressure overload and reduced cMLCK expression in RV myocytes. Representative line scans of Ca^2+^ spark recordings are shown from CTL (Figure 6a) and PAB (Figure 6b) RV myocytes. The frequency of Ca^2+^ sparks was significantly increased in PAB RV myocytes compared to CTL cells (Figure 6c). Other Ca^2+^ spark parameters including spark amplitude (Figure 6d), full width at half maximum (Figure 6e) and full duration at half maximum (Figure 6f) where not significantly different between CLT and PAB RV myocytes.

**FIGURE 7:**
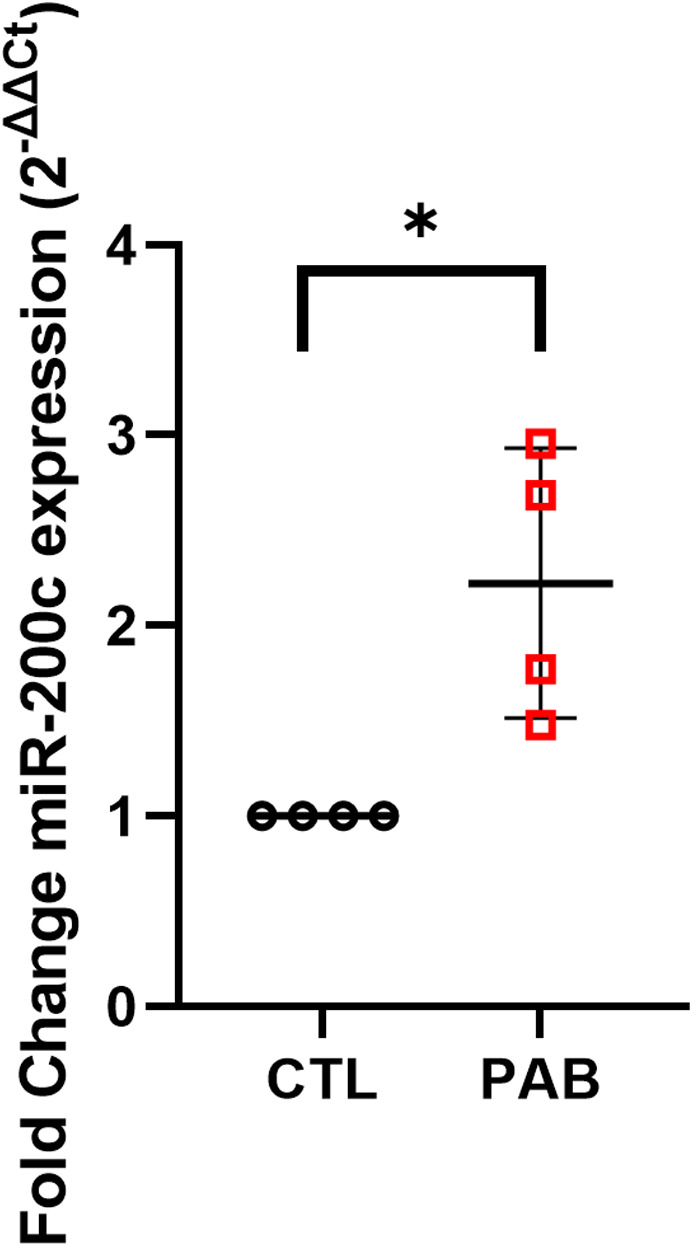
miR‐200c is upregulated in PAB RV myocytes compared to CTL. Real time RT‐PCR miRNA expression data for miR‐200c from CTL versus PAB RV myocytes normalized to U6 snRNA (*p* = 0.01, unpaired *t*‐test) (*n* = 4 CTL, *n* = 4 PAB) (*p < 0.05, unpaired *t* test)

Previous studies have implicated both microRNAs (miRs) and the ubiquitin‐proteasome machinery in the regulation of cMLCK expression in LV pressure‐overload (Hu et al., 2019; Warren et al., 2012). Our data presented in Figure 1 show that cMLCK mRNA and protein levels are both decreased in response to pressure‐overload of the RV compared to control. Recently, miR‐200c was shown to target cMLCK (*mykl3*) in LV pressure‐overloaded rats and cultured cells (Hu et al., 2019). miR‐200c levels in the heart have been shown to be increased by cellular reactive oxygen species, and pressure overload‐induced RV failure is characterized by increased oxidative stress (Hwang et al., 2021; Reddy & Bernstein, 2015; Sutendra et al., 2013). Given this link between RV pressure‐overload, ROS production, and miR‐200c expression, we focused on miR‐200c in our model of RV pressure‐overload. RT‐PCR analysis of miR‐200c levels showed that PAB RV myocytes have significantly higher expression of miR‐200c compared to CTL RV myocytes (Figure 7). These data indicate that miR‐200c may play a role in regulating the expression of cMLCK (*mylk3*) in the RV in response to pressure overload.

## 4 DISCUSSION

In this study, we aimed to identify the changes in RV cardiac myocyte protein expression as a result of pressure overload. To enrich for myocyte‐specific proteins, cardiac myocytes were isolated and purified from sham operated control (CTL) and pulmonary artery banded (PAB) right ventricles and subjected to mass spectrometry analysis 4‐weeks post‐surgery. Our results presented here show for the first time that cMLCK protein is downregulated in RV myocytes in response to pressure overload and may represent a part of the early hypertrophic response in the RV. cMLCK is an abundantly expressed protein that is critical for normal contractile function of cardiac myocytes and loss of cMLCK has been shown to play a role in the development of left ventricular hypertrophy (Chan et al., 2008; Gu et al., 2010; Hu et al., 2019; Massengill et al., 2016; Warren et al., 2012). Single myocyte contractility measurements showed that loss of cMLCK leads to impaired contraction and relaxation of RV myocytes. We also observed impaired diastolic calcium handling in the form of increased Ca^2+^ sparks and decreased Ca^2+^ transient decay kinetics in the PAB myocytes. Finally, we identified miR‐200c as upregulated in the RV in response to pressure overload that may control expression of cMLCK. These data provide novel insights to the maladaptation of the RV to pressure overload and present new targets for therapies aimed at restoring RV function. These data are especially critical for the congenital heart defect population, where right ventricle (RV) pressure overload remains in some patients after surgical correction of the defects and eventually impairs RV function and influences long‐term mortality and morbidity due to the limited treatment options are available.

cMLCK is activated by Ca^2+^/Calmodulin and leads to phosphorylation of myosin light chain 2 (MLC2) at Ser15 (Levine et al., 1996; Seguchi et al., 2007). In cardiac myocytes, this results in potentiation of both the rate and force of contraction. Reduced phosphorylation of MLC2v has been implicated in human heart disease where phosphorylation was reduced to ~18% of total MLC2v in failing hearts from ~30%–40% in healthy hearts (Velden et al., 2003). It was recently reported that cMLCK expression is higher in the right ventricle than in the left ventricle, indicating a more prominent role for cMLCK in regulating right ventricular contractility (Warren et al., 2012). Our results presented here are in support of this notion. Furthermore, we show for the first time that loss of cMLCK in the right ventricle leads to many of the similar contractility defects seen when cMLCK expression is reduced in the left ventricle (Chan et al., 2008; Gu et al., 2010; Massengill et al., 2016; Warren et al., 2012). For example, we observed significant dysfunction in both the contraction and relaxation of PAB RV myocytes similar to findings with failing LV myocytes. The recapitulation of the PAB phenotype in CTL myocytes with ML‐7 treatment, along with a lack of effect of ML‐7 on PAB myocytes, implicates the loss of cMLCK as a factor in the impaired contractility of PAB myocytes. A recent multiscale computation study has shown that impaired myocyte myofilament contractility is the major determinant of RV function in the pressure‐overloaded RV (Philip et al., 2018). Our data presented here, together with previous studies showing the importance of myocyte contractility to overall RV function in pressure‐overload and the increased expression level of cMLCK in the right ventricle, make this pathway a very promising target for the treatment of pressure‐overload induced RV hypertrophy and failure.

In addition to impaired contractility, we also observed dysregulated Ca^2+^ handling in PAB RV myocytes. PAB RV myocytes exhibited decreased Ca^2+^ transient amplitudes and slower decay times of the Ca^2+^ transient compared to control myocytes. The t‐tubule system was not significantly remodeled PAB RV myocytes compared to controls as has been reported in many models of LV failure (Guo et al., 2013; Ohler et al., 2009; Song et al., 2006; Wei et al., 2010) and in a monocrotaline‐induced model of pulmonary hypertension and RV failure (Xie et al., 2012). Consistent with this finding, we did not observe dysfunction in the Ca^2+^ transient rise kinetics in PAB RV myocytes. We also observed significantly more Ca^2+^ sparks in PAB RV myocytes compared to control myocytes. This increased diastolic leak could explain both the decreased Ca^2+^ transient amplitudes (due to decreased SR Ca^2+^ content) and the slower Ca^2+^ transient decay times. Overall, our data indicate that the Ca^2+^ release mechanism is dysregulated during diastole and the loss of cMLCK in the pressure‐overloaded RV contributes to the impaired contraction and relaxation of cardiac myocytes. While our Ca^2+^ handling data do not indicate any dysfunction in Ca^2+^ release kinetics, the increased diastolic Ca^2+^ leak in the form of Ca^2+^ sparks could also contribute to slowing both the Ca^2+^ transient decay kinetics and the myofilament relaxation kinetics. However, as we do not see any impairment in Ca^2+^ release kinetics, we hypothesize that the loss of cMLCK is responsible for the slowed contraction kinetics in PAB RV myocytes. While impaired SERCA function, expression, and regulation by phospholamban could also contribute to decreased SR Ca^2+^ content and slower Ca^2+^ transient decay times, we did not see significant changes in SERCA expression in PAB RV myocytes via mass spectrometry. Finally, the relative contributions of decreased cMLCK levels and reduced Ca^2+^ transient amplitude to the decreased sarcomere shortening in PAB RV myocytes are still unclear; however, we observed decreased sarcomere shortening in CTL cells when cMLCK was inhibited and did not see changes in Ca^2+^ transient amplitudes in these cells (data not shown). Overall, these findings warrant further examination of the mechanisms of dysregulated Ca^2+^ handling in PAB RV myocytes.

In previous studies, RV hypertrophy and failure as a result of pressure‐overload were characterized by decreased transcription and activity of electron transport chain complexes, altered mitochondrial function, and increased oxidative stress (Hwang et al., 2021; Reddy & Bernstein, 2015; Sutendra et al., 2013). Previous studies have shown a link between reactive oxygen species accumulation and miR‐200c expression (Climent et al., 2020; Magenta et al., 2017). Recently, miR‐200c was shown to be upregulated in response to reactive oxygen species accumulation in a model of cardiac hypertrophy, providing a role for miR‐200c in ROS‐mediated cardiac dysfunction (Hu et al., 2019). In our study, we provide preliminary indirect correlational data suggesting that miR‐200c may also be involved in the response of the RV to pressure‐overload. We found that *mylk3* mRNA was significantly downregulated and miR‐200c was significantly upregulated in response to pressure‐overload in the RV, however, whether miR‐200c is directly responsible for the decreased levels of *mylk3* is yet to be determined. Further studies are needed to fully understand the relationship of miR‐200c and *mylk3*/cMLCK expression in RV pressure overload. Overall, these data provide a potential link between the metabolic dysregulation and impaired cardiac myocyte contractility seen in RV failure and provide sights to the underlying cellular mechanisms leading to RV failure due to congenital heart defects (CHDs). Of interesting note, the *mylk3* gene is regulated by the cardiac homeobox protein Nkx2‐5 which is involved in the pathology of many CHDs (Chan et al., 2008). Whether dysfunction of Nkx2‐5 makes CHD patients more susceptible to pressure‐overload injury is still unknown.

In conclusion, our results indicate the loss of cMLCK is a critical maladaptation and early hypertrophic response of the RV to pressure overload and represents a novel target for therapeutic approaches to treat RV hypertrophy and failure associated with congenital heart defects. Improved understanding of the mechanisms of cardiac myocyte dysfunction in RV pressure overload will aid in the development of novel therapeutic targets to improve contractility and preserve long‐term RV function.

## CONFLICT OF INTEREST

None declared.

## AUTHOR CONTRIBUTIONS

V.P., N.M., and J.T.M conceived and planned the experiments. P.B. performed the mass spec analysis. V.P., N.M., R.R., A.P., B.M., R.K., J.R., J.D., and J.T.M. performed the experiments and analyzed the data. V.P., N.M., and J.T.M. wrote the manuscript and created the figures. The final version of the manuscript was approved by all authors.

## Supporting information



Fig S1

Fig S2

## Data Availability

The data that support the findings of this study are openly available at the ProteomeXchange Consortium via the PRIDE partner repository with the dataset identifier PXD027427 and are available from the corresponding author, J.T.M., upon reasonable request.
