# Supplementary figures and images for: Loss of cardiac myosin light chain kinase contributes to contractile dysfunction in right ventricular pressure overload
Source: Physiol Rep. 2022 Apr 5;10(7):e15238. doi: 10.14814/phy2.15238 (PMC8981447; doi:10.14814/phy2.15238)

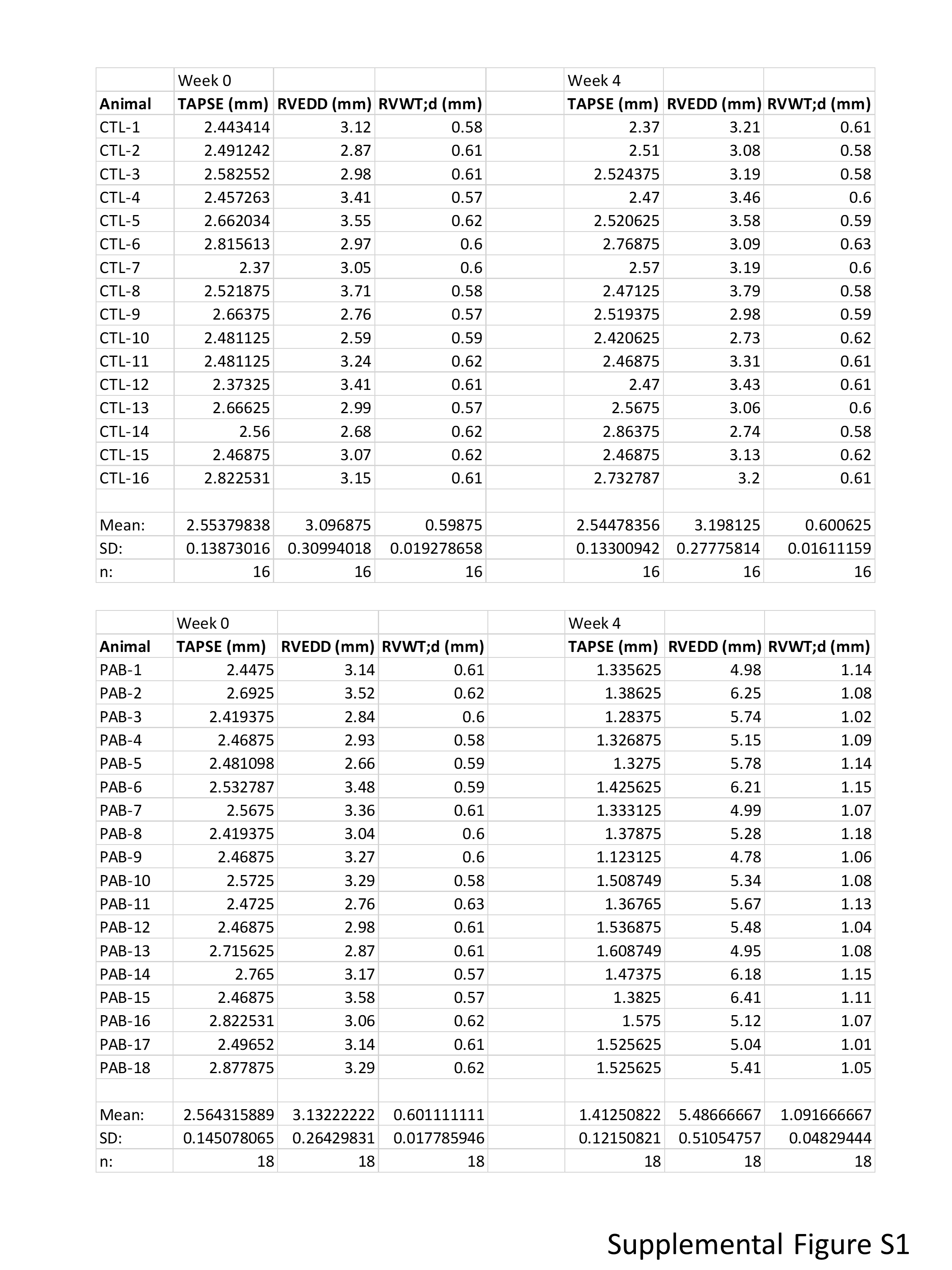

Supplement: Supplementary file 1 — Fig S1 [file PHY2-10-e15238-s001.tif]

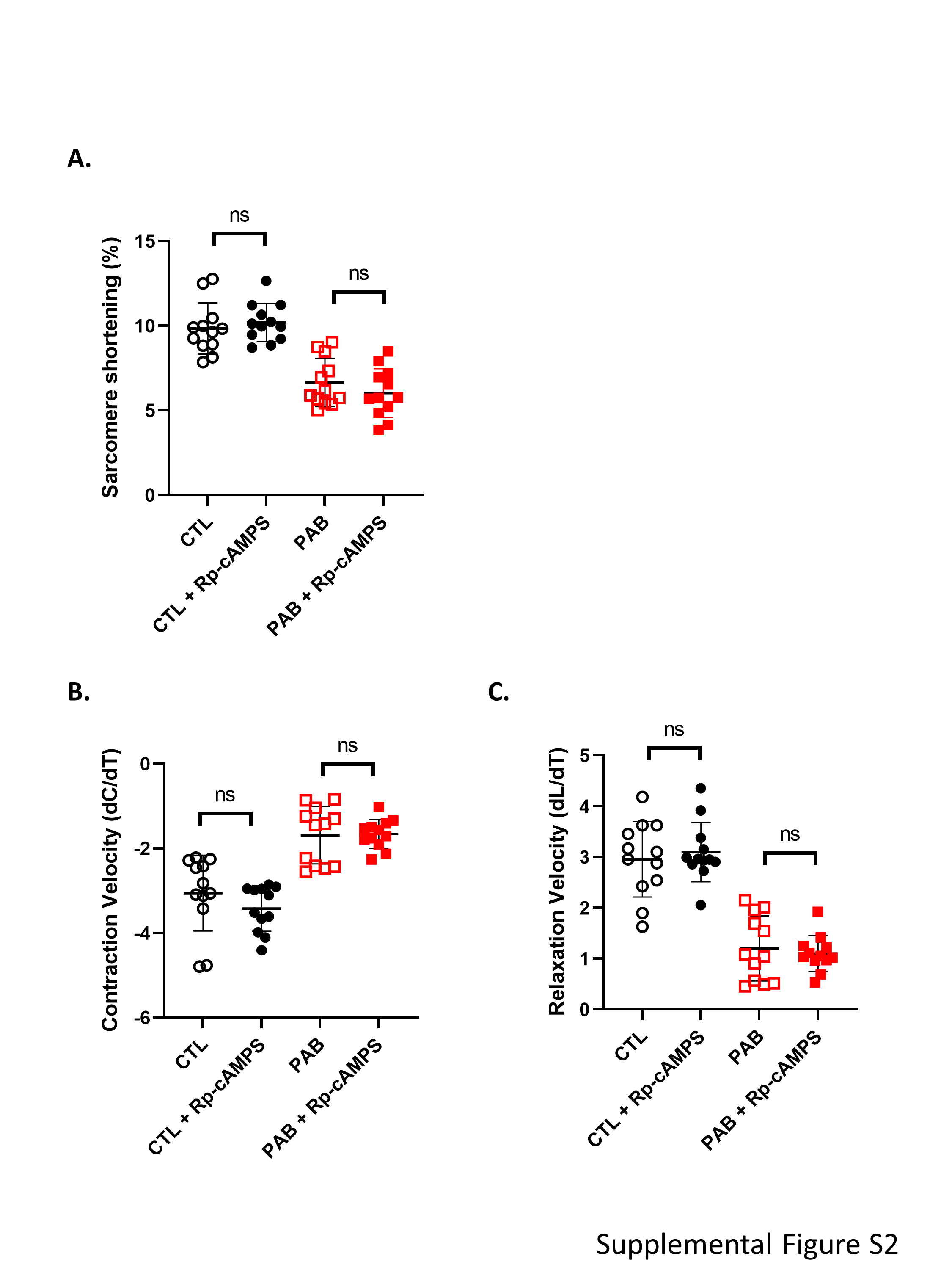

Supplement: Supplementary file 2 — Fig S2 [file PHY2-10-e15238-s002.tif]
